# Silencing of the Slt2-Type MAP Kinase *Bmp3* in *Botrytis* *cinerea* by Application of Exogenous dsRNA Affects Fungal Growth and Virulence on *Lactuca sativa*

**DOI:** 10.3390/ijms22105362

**Published:** 2021-05-19

**Authors:** Maria Spada, Claudio Pugliesi, Marco Fambrini, Susanna Pecchia

**Affiliations:** 1 Department of Agriculture Food and Environment, University of Pisa, Via del Borghetto 80, 56124 Pisa, Italy; claudio.pugliesi@unipi.it (C.P.); marco.fambrini@unipi.it (M.F.); 2 Interdepartmental Research Center Nutrafood “Nutraceuticals and Food for Health”, University of Pisa, Via del Borghetto 80, 56124 Pisa, Italy

**Keywords:** gray mold, lettuce, plant protection, topical application of dsRNA, gene knockdown, RNAi-based fungicides

## Abstract

*Botrytis cinerea* can attack over 500 genera of vascular plants and is considered the second phytopathogen in the ‘top ten’ for its economic importance. Traditional fungicides can be ineffective and with increasing fungicide resistance, new sustainable technologies are required. Lately, RNA interference-based fungicides are emerging for their potential uses in crop protection. Therefore, we assessed the potential of this innovative approach targeting the MAP kinase *Bmp3* in *B. cinerea*, a gene involved in saprophytic growth, response to low osmolarity, conidiation, surface sensing, host penetration and lesion formation. After performing a prediction analysis of small interfering RNAs, a 427 nucleotides long dsRNA was selected as construct. We tested the effect of topical applications of dsRNA construct both in vitro by a fungal growth assay in microtiter plates and in vivo on detached lettuce leaves artificially inoculated. In both cases, topical applications of dsRNA led to gene knockdown with a delay in conidial germination, an evident growth retardation and a strong reduction of necrotic lesions on leaves. These results correlated with a strongly reduced expression of *Bmp3* gene. In accordance to these findings, the *Bmp3* gene could be a promising target for the development of an RNAi-based fungicide against *B. cinerea*.

## 1. Introduction

The necrotrophic plant pathogenic fungus *Botrytis cinerea* Persoon: Fries [teleomorph *Botryotinia fuckeliana* (de Bary) Whetzel], responsible for the gray mold disease, can attack 586 genera of vascular plants [1] and ranks second in the Top 10 fungal plant pathogen list drawn up on the basis of its scientific and economic importance [2]. The pathogen has a huge economic impact on many important crops, particularly fresh fruits and vegetables, at both pre- and post-harvest stages. The pathogen has been estimated to cause annual losses of $10 billion to $100 billion [3] and fungicides that specifically target *B. cinerea* represent about 10% of the global fungicide market [4].

The control of *B. cinerea* is rather difficult due to its wide host range and environmental persistence and to date, the most common strategy for controlling *B. cinerea* is by chemical means. However, the use of conventional fungicides can cause the development of resistant strains and poses risks to human health and the environment [5].

Given the importance of *B. cinerea* in agricultural production, its control is of great relevance and new biotechnological strategies are ongoing to develop a more reliable and environmentally friendly management of gray mold diseases.

RNA interference (RNAi) based technology has proven to be a powerful tool that can be exploited to improve crop production and protection. RNAi is a process of Post-Transcriptional Gene Silencing (PTGS) triggered by double-stranded RNA (dsRNA), small interfering RNA (siRNA) or hairpin RNA (hpRNA), resulting in specific degradation of target mRNA.

In particular, the exogenous application of dsRNA-mediated RNAi has been reported as a non-Genetically Modified Organism (non-GMO) promising strategy in plant defense against some phytopathogenic fungi targeting specific genes. This innovative approach, called Spray-Induced Gene Silencing (SIGS), has been described to be efficient, potentially sustainable and environmentally friendly [6].

Topical or spray applications of dsRNAs and/or siRNAs have been demonstrated to be effective in the control of *Fusarium graminearum* in barley targeting genes involved in ergosterol biosynthesis [7] and of *F. asiaticum* in wheat targeting the *myosine 5* gene [8].

Uptake of RNAs from the environment, a phenomenon named environmental RNAi, was demonstrated in *B. cinerea* that can take up both siRNAs and dsRNAs directly, inducing silencing of the pathogen genes. The application of siRNAs or dsRNAs targeting *B. cinerea* Dicer-Like protein 1 (*Bc*-DCL1) and *Bc*-DCL2 on the surface of fruits, vegetables and flowers significantly reduced the gray mold disease. Moreover, sRNAs generated from the host plant cells are also transferred into *B. cinerea* cells, demonstrating a bidirectional cross-kingdom RNAi and sRNA trafficking between a plant host and a fungal pathogen [9].

McLaughlin et al. (2018) [10] reported that the topical application of dsRNA (single transcript) on detached leaves of *Brassica napus* significantly decreased disease symptoms and in some cases also disease severity caused by *Sclerotinia sclerotiorum* and *B. cinerea*.

Recently, three essential *B. cinerea* genes: *lanosterol 14α-demethylase*, *chitin synthase 1* and *elongation factor 2* (*BcCYP51, Bcchs1, BcEF2),* were used for in vitro and in vivo experiments in grapevine plants to confer protection against *B. cinerea* in both pre- and post-harvest conditions [11].

The infection process of *B. cinerea* involves stages of conidial attachment, germination, host penetration, primary lesion formation, lesion expansion and tissue maceration followed by sporulation [12]. Gene knockout approaches allowed the identification of genes involved in the different stages of disease cycle [13] and among them there could be possible target for the development of RNAi-based fungicides [14].

In recent years, many signaling factors have been identified as being involved in disease development, including mitogen-activated protein kinases (MAPKs) [15]. Signal transduction cascades regulating fungal development and virulence are remarkably conserved between distantly related fungi, especially those involving mitogen-activated protein kinases (MAPKs) [16]. Previous studies evidenced that a key player involved in the pathogenic signaling of *B. cinerea*-infected plants is represented by the Slt2-type MAP kinase *Bmp3* gene (orthologous to yeast *Slt2*). The Slt2-type MAP kinases are responsible for cell wall integrity in many filamentous fungi and various aspects of saprotrophic and pathogenic growth. Deletion of the *Bmp3* gene do not affected cell wall integrity as expected from the data on other fungi. The phenotype suggests that *Bmp3* is involved in saprophytic growth, response to low osmolarity, conidiation, surface sensing, host penetration and lesion formation. For these reasons, the Bmp3 MAP kinase presents unique features in *B. cinerea* [17,18].

The aim of this study was to assess the effectiveness of exogenous application of a *Bmp3*-targeting dsRNA construct in the silencing of *B. cinerea Bmp3* gene in vitro and in vivo on lettuce (*Lactuca sativa* L.) leaves.

## 2. Results

### 2.1. In Vitro Silencing of Bmp3 Gene Affects Growth and Morphological Traits of B. cinerea

**Figure 1:**
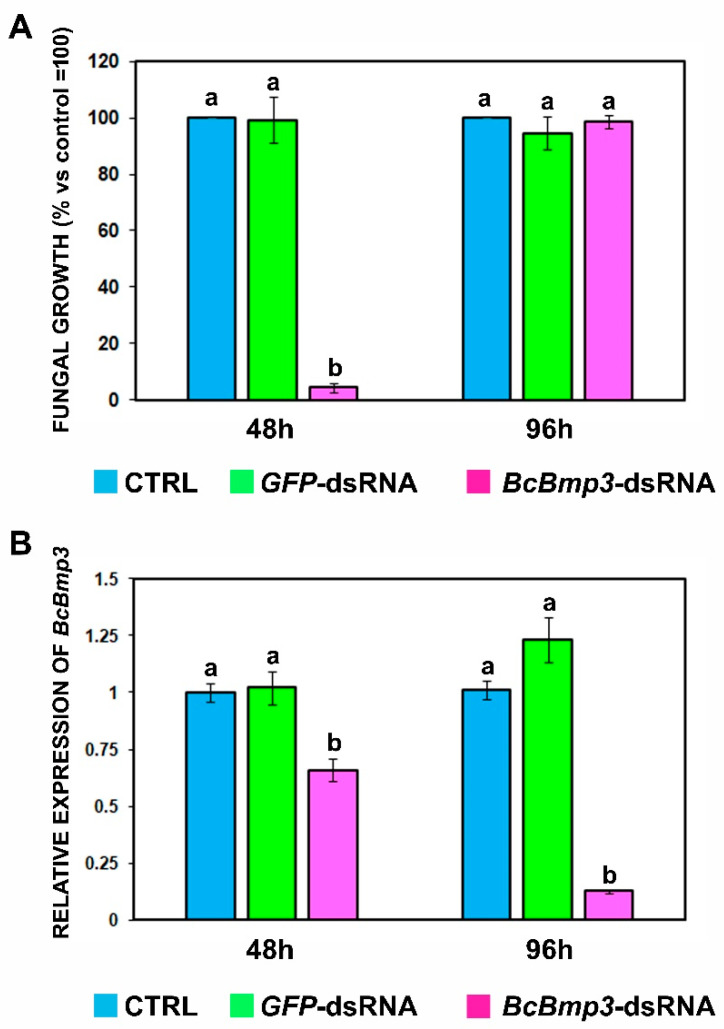
In vitro effects of *BcBmp3*-derived dsRNA on fungal growth. (**A**) Fungal growth was assessed measuring the optical density (OD_595_) at 48 and 96 h (48 h and 96 h) in 96-well microtiter plates. Each well contained 500 conidia of *B. cinerea* B05.10 in SMB medium and 2 μg of *GFP*-dsRNA (green) or *BcBmp3*-dsRNA (fuchsia). SMB + TE buffer was used as control (CTRL, blue). The graph shows the mean (±SE) of two independent experiments with eight biological replicates (*n* = 8). Same letters above the bars indicate no significant differences from each other (ANOVA) according to Tukey’s test (*p* ≤ 0.05). (**B**) Expression of *BcBmp3* mRNA in mycelium of *B. cinerea* B05.10 treated with *GFP*-dsRNA (green) or *BcBmp3*-dsRNA (fuchsia) at 48 and 96 h (48 h and 96 h). SMB + TE buffer was used as control (CTRL, blue). Relative transcript values were calculated by qRT-PCR using CTRL and *GFP*-dsRNA as reference samples and normalized to *BctubA* gene. The graph shows the mean (±SE) of three biological replicates (*n* = 3). Same letters above the bars indicate no significant differences from each other (ANOVA) according to Tukey’s test (*p* ≤ 0.05). The statistical analysis was conducted separately for 48 and 96 h.

To investigate whether knockdown of the *Bmp3* gene could affect the growth of *B. cinerea* in axenic culture, we generated a 427 bp dsRNA (*BcBmp3*-dsRNA), which was complementary to a portion of the fifth exon of the gene (Figures S1 and S2). To quantify the effects of *BcBmp3*-dsRNA on fungal growth, the optical density of fungal mycelium at different times was measured. The fungal growth was significantly delayed at 24 (1.89%), 48 (4.4%) and 72 h (67.5%) compared to the controls (CTRL = SMB + TE buffer and *GFP*-dsRNA) and was subsequently restored at 96 h (98.5%) (Figure S3 and Figure 1A). According to these results, two times 48 and 96 h were chosen to sample the mycelium in order to verify a correlation between reduced fungal growth and silencing of the target gene.

The expression of the *BcBmp3* gene was assessed by quantitative Real-Time PCR (qRT-PCR). After 48 h of incubation in the presence of *BcBmp3*-dsRNA, the expression of *BcBmp3* gene in the mycelium was significantly suppressed (63.6%) compared to both control media (Figure 1B). Notably, a more outstanding reduction in *BcBmp3* mRNA levels was detected 96 h after treatment: 12.6% vs. CTRL and 10.3% vs. *GFP*-dsRNA (Figure 1B). These data suggested that *BcBmp3*-dsRNA, delivered in vitro, silenced the expression of *BcBmp3* gene during the vegetative growth of the pathogen.

**Figure 2:**
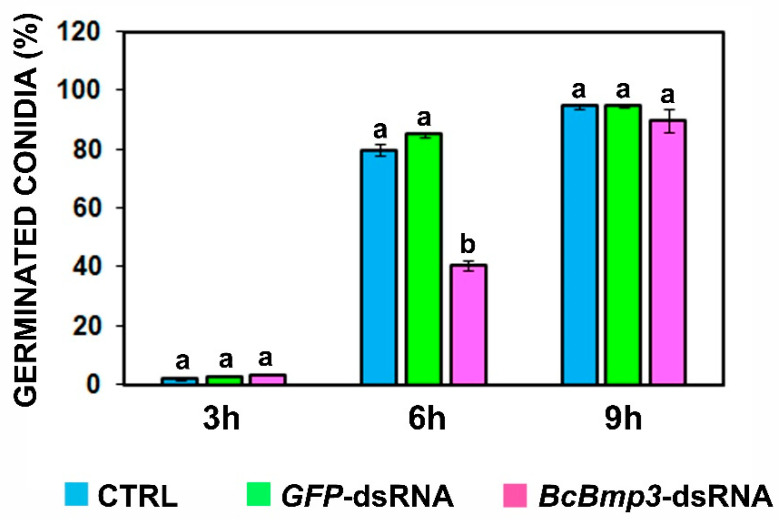
Germination kinetics of *B. cinerea* B05.10 conidia (1 × 10^6^) on liquid SMB in 96-well polystyrene microtiter plates in the presence of *BcBmp3*-dsRNA (fuchsia) or *GFP*-dsRNA (green) and control (CTRL = SMB + TE buffer, blue). The graph shows the mean (±SE) of two independent experiments with three biological replicates (*n* = 3; 200 conidia for each biological replicate were counted). Same letters above the bars indicate no significant differences from each other (ANOVA) according to Tukey’s test (*p* ≤ 0.05). The statistical analysis was conducted separately for 3, 6 and 9 h.

The effects of *BcBmp3*-dsRNA on kinetics of *B. cinerea* conidia germination were determined at 0, 3, 6 and 9 h on liquid SMB in 96-well polystyrene microtiter plates. The germination of conidia was significantly delayed at 6 h compared to the controls (CTRL = SMB + TE buffer and *GFP*-dsRNA). On average, the reduction in germination is about 50% (51% vs. CTRL and 48% vs. *GFP*-dsRNA). At 3 and 9 h, there were no significant differences between the treatments (Figure 2).

Conidia germination in the presence of *BcBmp3*-dsRNA, or controls (CTRL = SMB + TE buffer and *GFP*-dsRNA) was observed microscopically. At 0 h, non-germinated conidia showed the typical morphological features. They were globose or ellipsoidal, hyaline or pale-brown, usually with a protuberant hilum. After 3 h, *B. cinerea* conidia swelled rapidly and increased adhesion to one another (pre germination swelling). On average, 0 h conidia showed a volume of 441.2 ± 39.9 μm^3^ (mean size of the conidia: L 13.02 ± 0.24 × W 7.76 ± 0.50 μm). After 3 h, the mean volume of conidia (728.7 ± 33.0 μm^3^) increased by approximately 39.5% across all treatments.

**Figure 3:**
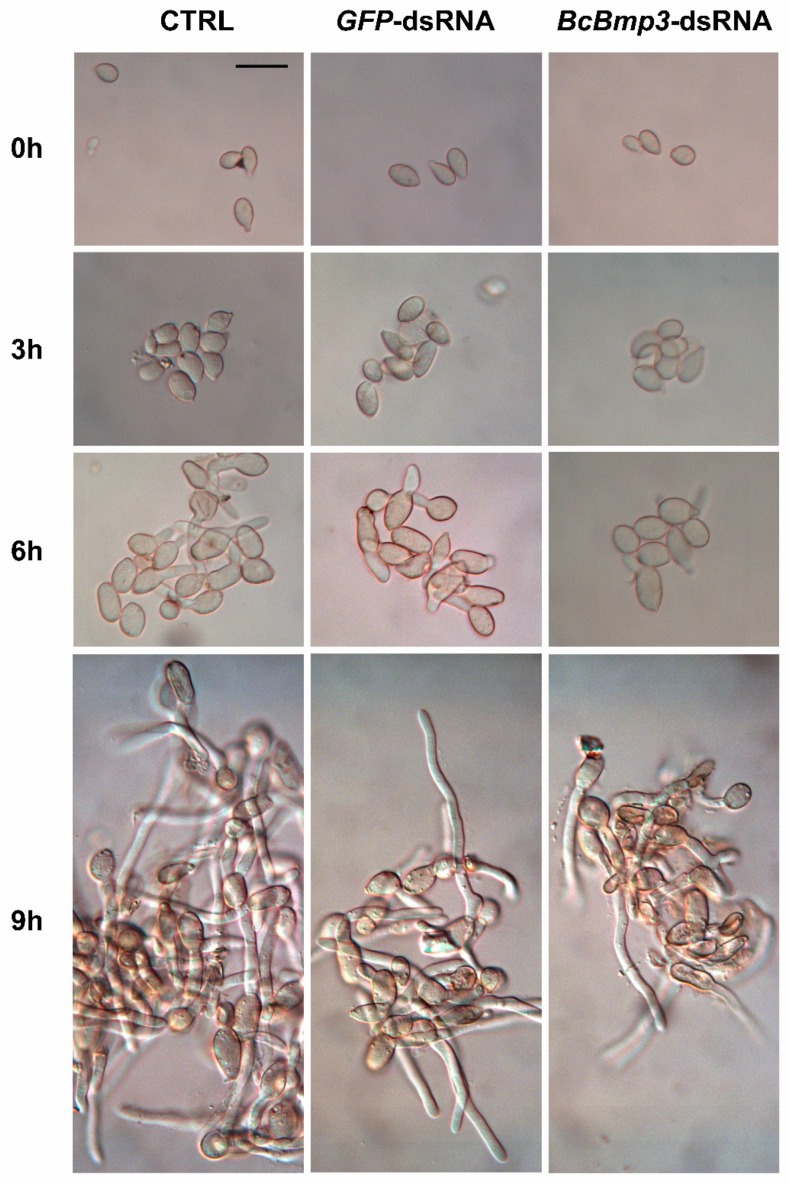
Germination of *B. cinerea* B05.10 conidia at 0, 3, 6 and 9 h of incubation in the presence of *BcBmp3*-dsRNA, or controls (CTRL = SMB + TE buffer and *GFP*-dsRNA). Scale bar = 20 μm applies to all subfigures.

The conidia after 6 h germinated forming one germ tube (sometimes two on opposite sides) with a germination rate of about 82% (79.7% vs. CTRL and 85.0% vs. *GFP*-dsRNA) whereas the germination rate in conidia treated with *BcBmp3*-dsRNA was 40.7%. After 9 h, germ tubes growth and elongation (>40 μm) increased progressively without differences in the germination rate between the treatments (Figure 3).

**Figure 4:**
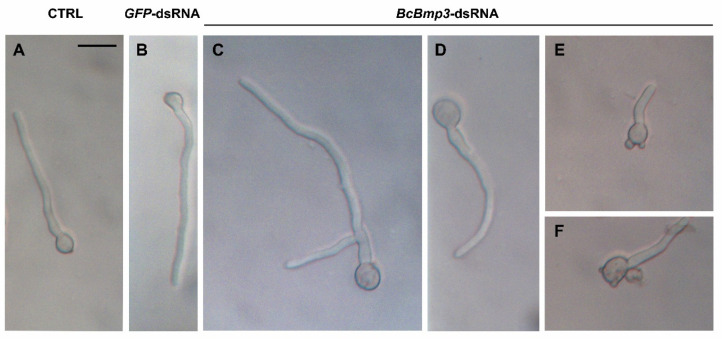
Pattern of germination of *B. cinerea* B05.10 conidia at 9 h of incubation in the presence of controls [CTRL = SMB + TE buffer, (**A**) and *GFP*-dsRNA (**B**)] or *BcBmp3*-dsRNA (**C**–**F**). Scale bar = 20 μm applies to all subfigures.

Moreover, in the controls, germ tubes grew more or less straight and growth appeared more directed and without alterations compared to the treated sample (Figure 4A,B). Conversely, when the conidia were treated with *BcBmp3*-dsRNA an altered mode of germination was often observed. The first branching events occurred near the conidium (Figure 4C), germ tubes grew more curved (Figure 4D) and some knobs were visible on the conidia surface (Figure 4E,F).

### 2.2. Bmp3 Expression is Reduced in Inoculated Lettuce Leaves Treated with BcBmp3-dsRNA

In vitro studies showed that *BcBmp3*-dsRNA could reduce the level of transcripts in *B. cinerea* for at least 96 h (4 dpi = days post inoculation) using a concentration of 20 ng μL^−1^ and 500 conidia. Starting from these observations, we assessed the effectiveness of the treatment with *BcBmp3*-dsRNA in reducing the symptoms caused by *B. cinerea* and the level of transcripts on *Lactuca sativa* cv. Romana using a detached leaf assay. After a topical application of *BcBmp3*-dsRNA (20 ng μL^−1^), leaves were inoculated with a drop of conidial suspension (500 conidia). Necrotic areas (mm^2^) were recorded and analyzed after 120 h (5 dpi).

**Figure 5:**
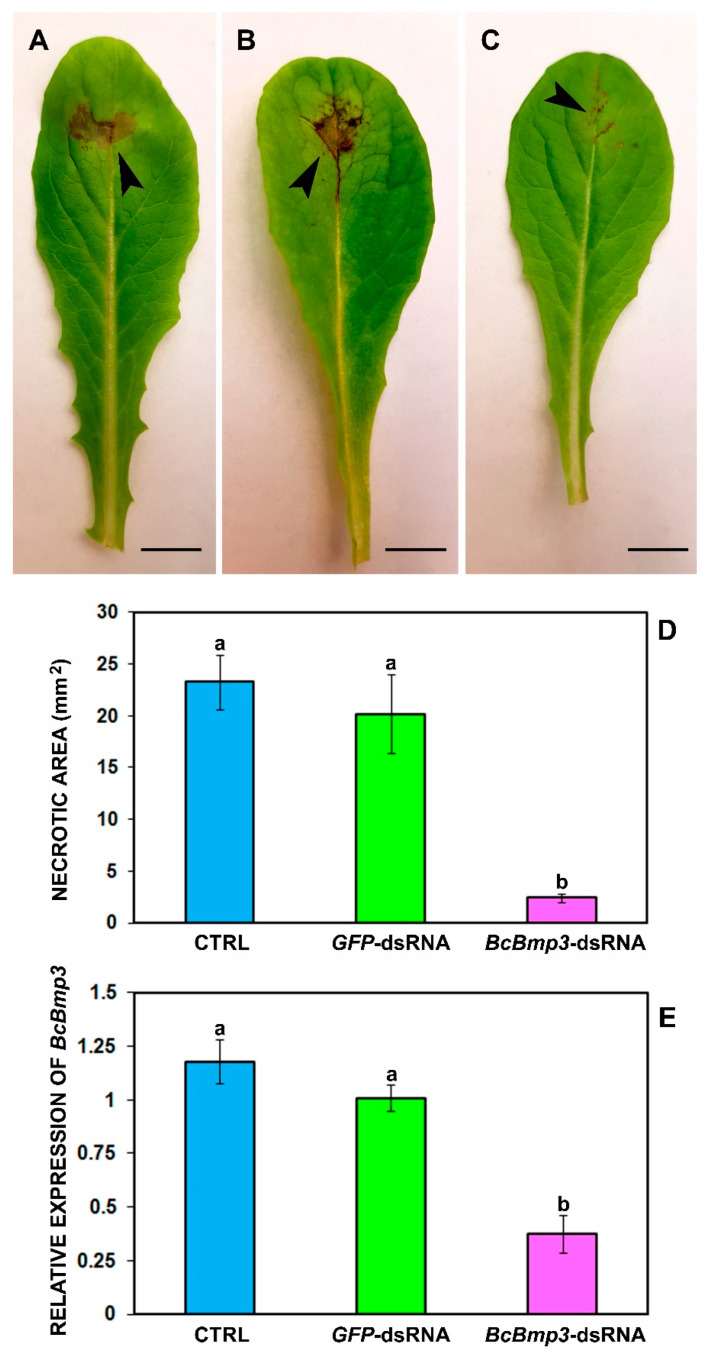
Infection symptoms of *B. cinerea* B05.10 on leaves of *Lactuca sativa* cv. Romana at 5 dpi. (**A**) Leaves were treated with water + TE (CTRL), (**B**) GFP-dsRNA and (**C**) *BcBmp3*-dsRNA and then were artificially inoculated with 5 µL of a conidial suspension (500 conidia) of the pathogen. (**D**) The necrotic areas (in mm^2^) using the ImageJ software Version 1.53a were measured. The graph shows the mean (±SE) values of two independent experiments with sixteen biological replicates (n = 16). (**E**) Transcription of *BcBmp3* mRNA at 5 dpi. Relative transcript values were calculated by qRT-PCR using *GFP*-dsRNA and CTRL as reference samples and normalized to *BctubA* gene. The graph shows the mean (±SE) values of three biological replicates (*n* = 3). Same letters above the bars indicate no significant differences from each other (ANOVA) according to Tukey’s test (*p* ≤ 0.05). Scale bars = A: 8.8 mm; B: 8.9 mm; C: 8.4 mm.

At 5 dpi control leaves treated with both water +TE and *GFP*-dsRNA (20 ng μL^−1^), showed necrotic lesions representing a successful *B. cinerea* infection on lettuce leaves (Figure 5A,B) whereas *BcBmp3*-dsRNA treated leaves showed strongly reduced necrotic lesions at inoculation site (Figure 5C).

Lettuce leaves treated with *BcBmp3*-dsRNA developed lesions about ten times smaller (2.4 ± 0.4 mm^2^) than control leaves treated with both water +TE buffer (23.3 ± 2.6 mm^2^) and *GFP*-dsRNA (20.2 ± 3.8 mm^2^) (Figure 5D).

To verify if the reduction of *B. cinerea* necrotic areas on *Bmp3*-dsRNA-treated leaves at 5 dpi correlated with a gene knockdown a qRT-PCR analysis was performed on inoculated leaves. The *Bmp3* transcript levels were drastically reduced: 31.8% and 37.2% compared to CTRL and *GFP*-dsRNA, respectively (Figure 5E).

### 2.3. BcBmp3-dsRNA: Off-Target Prediction and In Vitro Effects Against the Off-Target Fungus Trichoderma harzianum T6776

To explore co-silencing effects, we calculated possible off-targets for the tested *BcBmp3*-dsRNA construct. Precursor sequence of the construct was targeted against the complementary DNAs (cDNAs) of different phytopathogenic and beneficial fungi, human and lettuce using the si-Fi v21 software (default parameters). The results were summarized in Table S1 as the number of siRNA hits found (total and efficient) for the corresponding target.

In *Sclerotinia sclerotiorum* alone, 18 siRNAs were found, of which only 9 were efficient. The target sequence found (GenBank accession number EDO02968) corresponds to the catalytic domain of the serine/threonine kinase, fungal mitogen-activated protein kinase MPK1, also called Slt2. The sequence is the Stl2 ortholog *Smk3* of *S. sclerotiorum* similarly to the Stl2 ortholog *Bmp3* in the close relative *B. cinerea*. In this specific case, the results obtained by this bioinformatic analysis proved to be usefulness to guide the choice of multiple targets for RNAi.

Therefore, *BcBmp3*-dsRNA construct resulted highly specific for the target *Bmp3* gene of *B. cinerea* with no sequence off-target in the host plant (*Lactuca sativa*), distantly related phytopathogenic fungi (*Alternaria alternata*, *Fusarium oxysporum*, *Rhizoctonia solani*, *Pythium ultimum*), beneficial fungi (*Trichoderma asperellum*, *T. harzianum*, *Rhizophagous irregularis*) or human genome. Using the databases of three *B. cinerea* isolates, including B05.10, as expected, a high number of efficient off-target siRNA were found that exactly match only with the *Bmp3* gene (Table S1 and Figure S4).

To further explore co-silencing effects of the *BcBmp3*-dsRNA construct against off-targets organisms, we tested it against the promising biocontrol agent *T. harzianum* T6776. For this specific *T. harzianum* isolate the off-target prediction analysis with the si-Fi v21 software did not found any efficient siRNA (Table S1).

To quantify the effects of *BcBmp3*-dsRNA on fungal growth, the optical density (OD_595_) of fungal mycelium at different times (24, 48, 72 and 96 h) was measured. Growth of *T. harzianum* T6776 was not significantly reduced at any time compared to controls [CTRL = SMB + TE buffer and *GFP*-dsRNA] confirming both the high specificity of the construct and the prediction analysis (Figure S5).

## 3. Discussion

In this work, we demonstrate that the Slt2-type mitogen-activated protein kinase (MAPK) *Bmp3* gene is an efficient and novel target for RNAi with the purpose of reducing disease symptoms in lettuce after *B. cinerea* infection. The topical application of a *BcBmp3*-dsRNA construct mediated both in vitro and in vivo knockdown of the *B. cinerea* transcripts. To the best of our knowledge, this is the first report on the reduction of *B. cinerea* growth and virulence through a topical application of dsRNA targeting a MAPK gene.

The family of serine/threonine protein kinases known as mitogen-activated protein kinases (MAPKs) are involved in the transduction of a variety of extracellular signals and the regulation of different developmental processes.

The MAPK orthologs of yeast Slt2 are conserved in filamentous ascomycetes and they regulate cell wall integrity (CWI) and pathogenesis [19]. In addition to their conserved roles in infection and cell wall integrity, Slt2 orthologs are also involved in regulating different biological processes in plant pathogenic fungi [16]. The Slt2 pathway is primarily involved in pathogenicity, invasion, vegetative growth, conidiation, cell wall integrity, anastomosis and response to oxidative stress. In *B. cinerea*, *Bmp3* mutants are defective in surface sensing, plant penetration and induction of necrotic lesions [17]. By contrast, the deletion of this gene in *B. cinerea* has no obvious effect on cell wall sensitivity, thus evidencing that *Bmp3* presents unique features in this fungal pathogen [20].

For these reasons, we considered this gene a good potential target for RNAi mediated by the exogenous application of a dsRNA construct complementary to a region of the *B. cinerea Bmp3* sequence. Moreover, the target sequence was chosen so that in silico it shares no homology with the genes of the host and of other off-target organisms. Using the si-Fi v21 software, the *BcBmp3*-dsRNA construct resulted highly specific for *B. cinerea* with no off-target hits in the host plant, in distantly related phytopathogenic fungi and in beneficial fungi or human genome. Nine siRNAs were found to be efficient only in the close relative *Sclerotinia sclerotiorum*.

The choice of highly specific target sequences should avoid homology to off-target transcripts and minimize off-target impacts. Using specific software and databases, it is possible to test different regions of a gene minimizing off-target hits. Nevertheless, the best approach to reduce risks is to combine bioinformatics analyses with biological data [21]. In light of this, we tested *BcBmp3*-dsRNA construct against the promising biocontrol agent *T. harzianum* T6776, which did not show negative results either in silico or in vivo.

The first approach used to validate the candidate gene was to in vitro challenge the dsRNA molecule with the fungal pathogen. The *BcBmp3*-dsRNA construct was applied to liquid cultures of *B. cinerea*, which was grown in 96-well microtiter plates. This assay is considered very simple, cost-effective and rapid to quantify the inhibitory effects of molecules on fungal growth [22]. Furthermore, the assay can be very useful for a preliminary evaluation of the effects of dsRNA constructs on the vegetative growth and on the conidia germination of fungi using small amounts of dsRNA [23].

The validity of in vitro studies using dsRNA constructs against fungal pathogens has been demonstrated by different studies. Molecules have been designed to target essential genes in *Fusarium oxysporum* f.sp. *cubense* and *Mycosphaerella fjiensis* [24], *Sclerotinia sclerotiorum* [10] and *Fusarium graminearum* [23].

In this study, the applications of *BcBmp3*-dsRNA construct led to gene knockdown with an evident growth retardation, a delay in conidia germination and an altered morphology of germ tubes.

Comparing the expression data with the vegetative growth data, it can be highlighted that the strength of *BcBmp3* gene knockdown is not directly related to growth retardation in accordance with other studies [23]. Transcript levels at 48 h were significantly lower than controls using topical application of *BcBmp3*-dsRNA. Anyway, the strongest silencing of gene expression was observed at 96 h when vegetative growth was restored. In *S. sclerotiorum*, 48 h are required for optimal RNAi silencing using topical application of dsRNA and the level of suppression persisted at 96 h not significantly changing from 48 h [10].

Previous research has shown that *B. cinerea* knockout mutants in the *Bmp3* gene showed growth retardation, impaired conidiation and loss of sclerotia formation indicating that *Bmp3* is required for normal saprotrophic growth [17]. The Slt2 type MAPK *Bmp3* is involved in melanin biosynthesis [25] and qRT-PCR assays revealed that some chitin synthase-encoding genes (*CHS1*, *CHS3a*, *CHS5* and *CH6*) were down-regulated in the *B. cinerea* MAPK *Bmp3* mutant [26].

Slt2 homologs are well known for their crucial roles on growth in knockout mutants of different fungi and Oomycota. In *Alternaria alternata* fungal strains defective in *AaSlt2* displayed retardation in radial growth compared with the wild type and produced globose, swollen hyphae that did not elongate in a straight radial direction [27]. Silencing of a Slt2 type MAPK in the oomycete *Phytophthora sojae* showed defects in fungal growth, zoosporogenesis and increased hypersensitivity to cell-wall degrading enzymes [28].

In *Colletotrichum gloeosporioides,* a *Cgl-Slt2* mutant revealed a defect in vegetative growth and sporulation compared to the wild-type strain [29]. A growth defect has been also reported in Slt2-type MAPK mutants of *Fusarium graminearum* [30] and in transformants of *Pseudocercospora fijiensis* using RNAi-mediated gene silencing [31].

In *S. sclerotiorum*, a close relative of *B. cinerea*, disruption of the Slt2 ortholog *Smk3* gene caused loss of the ability to produce sclerotia, increased aerial hyphae formation, altered hyphal hydrophobicity and cell wall integrity, slowed radial expansion rates on solid media [32].

Germination of *B. cinerea* conidia was reduced by approximately 50% after 6 h of incubation in the presence of the *BcBmp3*-dsRNA construct compared to the controls. Furthermore, some germination defects after 9 h of incubation were also observed. Conversely, *B. cinerea* knockout mutants in the *Bmp3* gene showed normal germination rates and germ tubes showed excessive elongation [17].

However, some defects in conidia germination or hyphae development have been found in other pathogenic fungi treated with dsRNAs for RNAi. Synthetic dsRNAs showed high levels of spore germination inhibition of two major pathogenic fungi of banana: *Fusarium oxysporum* f. sp. *cubense* and *Mycosphaerella fijiensis* [24]. Abnormal branching of developing hyphae of *F. graminearum* grown in axenic culture following treatment with *CYP51*-dsRNAs was observed [23]. Microscopic analysis of conidia treated with *FaMyo*5-8 dsRNA revealed clear growth inhibition and abnormal mycelium in *Fusarium asiaticum* and other *Fusarium* species [8].

Germination rates and germ tube morphology are altered in a *B. cinerea Bem1* mutant. Defects induced by this mutation are very similar to that observed in our experiments. A phenotypic overlap with Δ*bcbem1* is evidenced for mutants lacking *Bmp3* [33].

Here we used the *Lactuca sativa* cv. Romana*-B. cinerea* pathosystem to evaluate the efficacy of the *BcBmp3*-dsRNA construct in reducing disease symptoms. We performed a detached leaf assay using as inoculum a conidial suspension of the pathogen after topically treating lettuce leaves with the dsRNA construct.

Gray mold caused by the fungus *B. cinerea* has been considered as a major disease in greenhouse-grown lettuce. The romaine lettuce variety and some iceberg lettuces are susceptible to *B. cinerea* also in the field [34].

The topical application of the *BcBmp3*-dsRNA construct reduced lesion areas at 5 dpi about ten-fold compared to controls. This strong decrease of necrotic areas was associated to a remarkably reduced level of *Bmp3* transcripts on infected leaves. Similarly, the external application of dsRNAs targeting *B. cinerea DCL1* and *DCL2* genes on the surface of fruits, vegetables and flower petals significantly inhibits gray mold disease [9]. Recently, it was demonstrated that SIGS application of dsRNAs can confer protection of grapevine against *B. cinerea* in both pre- and post-harvest conditions [11].

*B. cinerea* lesion area is correlated with the fraction of fungal RNA across lesion development stage. Thus, lesion area is a useful approximation of the interaction between the fungal and plant genetic variation [35].

Our findings are supported by previous studies, which demonstrated that *B. cinerea* knockout mutants in the *Bmp3* gene showed reduced penetration efficiency into primary lesion formation and expansion. In particular, it was highlighted that the growth of *B. cinerea* in host tissues appeared to be more strongly reduced than its growth in artificial media [17]. Moreover, *BcBmp3* gene revealed a delay in penetration of killed onion epidermis evidencing virulence defects [26].

Several studies using knockout mutants identified *Slt2* homologs as important genes in regulating virulence factors and pathogenicity in other fungal pathogens [36].

In *Pseudocercospora fijiensis*, the RNAi-mediated gene silencing of the *PfSlt2* gene evidenced that the pathogen showed less virulence characterized by reduced efficiency of plant infection, reduced invasive growth and fewer necrotic symptoms on banana plants [31]. The *Cgl-Slt2* MAP kinase in *Colletotrichum gloeosporioides* is required for conidiation, polarized growth, appressorium formation and pathogenicity [29].

The MAP kinase Slt2 homologs are essential for invasive growth of Mycosphaerella graminicola [37] and virulence in Alternaria alternata [27], Beauveria bassiana [38], Colletotrichum higginsianum [39], Fusarium graminearum [30], Phytophthora sojae [28] and Sclerotinia sclerotiorum [32].

Topical application of dsRNA molecules that triggers gene knockdown is a flexible approach that does not require transgenic plants for RNAi-based protection against plant diseases. However, some factors need to be overcome to get practical applications of this fascinating biotechnological approach. dsRNA molecules are susceptible to degradation when exposed in the environment, such as on the surface of plants or fruits. One of the best approaches to increase the stability and longevity of dsRNA for topical applications is to complex it with biocompatible nanoparticles [21]. Another factor to face is a cost-efficient dsRNAs production at large scale. About this, bacteria and yeasts now can be easily used to produce dsRNA molecules lowering significantly the production costs [40,41].

The basic knowledge obtained from our studies will contribute to the development of RNAi-based strategies against *B. cinerea*. SIGS experiments with naked dsRNA or dsRNA loaded onto nanoparticles are in progress.

## 4. Materials and Methods

### 4.1. Fungal Strains and Culture Conditions

*Botrytis cinerea* strain B05.10 is universally known as a haploid strain obtained after benomyl treatment of the wild-type isolate SAS56 [42,43].

In this study, *Trichoderma harzianum* T6776 was used as an off-target organism. This beneficial fungus is a promising biocontrol agent against different plant pathogens [44].

Both fungi were incubated on PDA (Biolife Italiana S.r.l., Milano, Italy) plates at 25 °C with a 12/12 NUV/light cycle unless indicated otherwise. The conidial suspensions of both fungi were prepared from 7 to 10-days-old PDA cultures by gently scraping conidia from the surface of the cultures with a sterile spatula in Sabouraud Maltose Broth [SMB: mycological peptone (Sigma-Aldrich, Saint Louis, MO, USA) 10 g L^−1^, maltose (Sigma-Aldrich, Saint Louis, MO, USA) 40 g L^−1^ pH 5.6 ± 0.2] prepared with MilliQ water (EASYpure® II LF, Thermo Scientific, Waltham, MA, USA; resistivity 18.2 MΩ cm^−1^). The resulting conidial suspensions were filtered through a layer of sterile Miracloth (Calbiochem, San Diego, CA, USA), conidia concentration was checked with a hemacytometer (Bürker, LO - Laboroptik Ltd, Lancing, UK) and adjusted to desired concentration with SMB.

### 4.2. In Vitro Effects of BcBmp3-Derived dsRNA on Growth and Conidia Germination of Botrytis cinerea

Fungal growth was studied in 96-well polystyrene microtiter plates (Cellstar^®^, Greiner Bio-One, Frickenhausen, Germany) in order to evaluate the effects of *Bmp3*-derived dsRNA (*BcBmp3*-dsRNA). Aliquots of a *B. cinerea* conidial suspension in SMB containing 500 conidia and 2 μg (final concentration 20 ng µL^−1^) of *Bmp3*-dsRNA were added to the wells (*n* = 8 for each treatment in a total assay volume of 100 μL).

Controls included in each plate were prepared in SMB and consisted of: (i) 2 μg of green fluorescent protein (GFP)-derived dsRNA (*GFP*-dsRNA) + 500 *B. cinerea* conidia, (ii) no dsRNA + 500 *B. cinerea* conidia + TE buffer (10 μM Tris/1.0 μM EDTA, pH 7.0), (iii) no dsRNA and no conidia + TE buffer (blank background control). The volume of TE corresponds exactly to the volume of dsRNA added. Plates were incubated at 25 °C with a 12/12 NUV/light cycle and fungal growth was assessed measuring the optical density (OD) at 595 nm with a microplate reader spectrophotometer (Bio-Rad, Model 680, Bio-Rad Laboratories, Cressier, Switzerland) at different times between 0 and 96 h. The experiment was performed twice.

After 48 and 96 h of incubation, *B. cinerea* mycelium was collected and washed with sterile MilliQ water by centrifugation for fungal transcript analysis to assess silencing of the *Bmp3* gene.

To determine the effect of *Bmp3*-dsRNA on conidia germination of *B. cinerea,* the experiments were performed as described before except in this case conidial suspension was adjusted to 1 × 10^6^ conidia for each well. After 0, 3, 6 and 9 h of incubation, germination rates were determined microscopically (Dialux 22, Leitz, Germany) by taking small aliquots from each well. Conidia *(n* ≥ 200 for each biological replicate) were counted as germinated when the germ tube length exceeded the conidial diameter. Images were captured using a Leica DFC 450C digital microscope camera with control software (Leica Microsystems Ltd., Heerbrugg, Switzerland). The percentage of germination was estimated by counting the number of germinated conidia relative to the total number of conidia. The conidia volume (V) was calculated according to the formula V = 4/3 · π · a/2 · b^2^/4 [45] by measuring length and width of the conidia (*n* ≥ 30). The experiment was performed in triplicate and repeated twice.

### 4.3. Botrytis cinerea Infections

*Lactuca sativa* cv. “Romana” (Romana Verde degli Ortolani, Sementi Dom Dotto S.p.A., Udine, Italy) plants were grown in a climate chamber with 12 h photoperiod and 22 ± 1 °C with 65% relative humidity.

Then, 20 μL of *BcBmp3*-dsRNA (20 ng µL^−1^), *GFP*-dsRNA (20 ng µL^−1^) or sterile MilliQ water + TE buffer (10 μM Tris/1.0 μM EDTA, pH 7.0) were dropped on the adaxial surface of detached lettuce leaves (third, fourth pair) from 3 to 4-weeks-old plants (*n* = 16). The volume of TE corresponds exactly to the volume of dsRNA added. The applications were allowed to dry and then droplets of 5 µL of the *B. cinerea* conidial suspension (500 conidia) were carefully placed on the same spot. Inoculated leaves were placed on sterile filter paper moistened with sterile MilliQ water in transparent plastic boxes (16 × 11.5 × 5 cm) and were incubated at 25 °C with a 12/12 NUV/light cycle.

Infection symptoms at 5 days post-inoculation (dpi) were observed and photographed. The necrotic areas (mm^2^) were measured using the ImageJ software Version 1.53a (http://imagej.nih.gov/ij, accessed on 13 January 2021) from digital images of the detached leaves [46]. The experiment was repeated twice.

### 4.4. DNA Extraction

For DNA extraction, fungal mycelium of *B. cinerea* B05.10 was grown in sterile 50 mL Falcon tubes filled with 25 mL of SMB at 150 rpm (Rosi1000™, Thermolyne, Dubuque, IA, USA) for 2–4 d at 25 ± 1 °C. The mycelium was harvested by filtration through a layer of sterile Miracloth, washed with sterile MilliQ water and dried on sterile filter paper. Total genomic DNA was extracted by the Genesig Easy DNA/RNA extraction kit (Primer Design Ltd., Chandler’s Ford, UK). The kit technology uses minute magnetic particles to bind DNA/RNA when they are placed onto a magnetic separator. The mycelium (200 mg) was placed into a 2 mL sterile extraction tube prefilled with 0.5 mm silica acid washed glass beads (Sigma-Aldrich, Saint Louis, MO, USA) and 500 μL of Sample Prep Solution supplied by the kit. The mycelium was then homogenized by a bead-beating method using the BeadBug™ Microtube homogenizer (Benchmark Scientific Inc., Sayreville, NJ, USA). Tubes were subjected to three beating cycles of 30 s at 4000 rpm followed by 30 s on ice. The lysed suspension (200 μL) was collected and DNA extraction was performed following the kit manufacturer’s instructions.

### 4.5. Sequence Analysis and Identification of BcBmp3 Template

In silico analysis using the si-Fi v21 software (siRNA Finder ver. siFi21_1.2.3-0008, https://sourceforge.net/projects/sifi21/, accessed in 15 January 2021), a program designed for RNAi silencing efficiency predictions and off-target analysis, was performed on the sequence of the *Bmp3* CDS of *Botrytis cinerea* B05.10 (GenBank accession number CP009813.1; Figure S1) in order to choose the optimal sequence for RNAi construct design (Figure S2). The default parameters were used selecting as database the cDNA of *B. cinerea* B05.10 obtained from EnsemblFungi (ftp://ftp.ensemblgenomes.org/pub/fungi/release-50/fasta/botrytis_cinerea/cdna/, accessed in 29 January 2021).

Genomic DNA of *B. cinerea* B05.10 was used as template for the synthesis of a partial sequence of the *mitogen-activated protein kinases* (*MAPK*) *Bmp3* gene (GenBank accession number CP009813.1) 427 bp long (Table S2). The PCR conditions were the following: 95 °C for 5 min, 5 cycles (30 s at 95 °C, 30 s at 61 °C, 30 s at 72 °C) and 30 cycles (30 s at 95 °C, 30 s at 76 °C, 30 s at 72 °C), 72 °C for 5 min. The pCT74-sGFP plasmid [47] was used as a template for the synthesis of *Green Fluorescent Protein* (*GFP*)-dsRNA (Table S2) of 712 bp. The PCR conditions were the following: 95 °C for 5 min, 5 cycles (30 s at 95 °C, 30 s at 55 °C, 30 s at 72 °C) and 30 cycles (30 s at 95 °C, 30 s at 76 °C, 30 s at 72 °C), 72 °C for 5 min. The Phusion High-Fidelity DNA polymerase was used (Thermo Fischer Scientific, Vilnius, Lithuania). The amplified products were purified and sequenced on both strands. The following software were used to analyze the amplified sequence: GENESCAN, FASTA, BLAST and CLUSTALW available at the National Center for Biotechnology Information (NCBI; http://www.ncbi.nlm.nih.gov/, 27 November 2020) [48]. Moreover, PROSITE and PFAM databases were used to identify conserved domains [49,50]. The conserved motifs were also recognized by searching the Conserved Domain Database (CDD) at NCBI and by the program InterPro at EMBL-EBI (http://www.ebi.ac.uk/, accessed on 3 December 2021). The analyses were performed using the representative genome of *Botrytis cinerea* B05.10 [51,52] and the *GFP* sequence of the pCT74-sGFP plasmid [47].

### 4.6. dsRNA Synthesis

dsRNAs were generated using MEGAscript RNAi Kit (Invitrogen by Thermo Fisher Scientific, Vilnius, Lithuania) following the MEGAscript protocol. Primer pairs for *BcBmp3*-dsRNA (Figure S1) and for *GFP*-dsRNA with T7 promoter sequence at the 5′ end of both forward and reverse primers were designed for amplification of dsRNA (Table S2).

### 4.7. Gene Expression Analysis by Quantitative Real-Time Polymerase Chain Reaction (qRT-PCR)

Total RNA was extracted from 100 mg of mycelium or lettuce leaves treated and untreated with *GFP*-dsRNA or *BcBmp3*-dsRNA as described above. The samples were collected from two independent experiments. Extraction was performed using the RNeasy Plant Mini Kit (Qiagen, Milan, Italy) following manufacturer’s instructions. The concentration of each RNA sample was measured using the Qubit^TM^ RNA BR Assay Kit in a Qubit™ 4 Fluorometer (Invitrogen by Thermo Fisher Scientific Inc., Eugene, OR, USA), and their integrity was evaluated by agarose gel electrophoresis. The RNA samples were treated with Amplification Grade DNase I (Sigma-Aldrich, St. Louis, MO, USA) and reverse-transcribed into cDNA (400 ng per sample) using iScript cDNA synthesis kit (BioRad, Hercules, CA, USA). The synthesized cDNA was used for quantitative Real-Time Polymerase Chain Reaction (qRT-PCR) using gene-specific primer pairs (Table S2). Real-Time quantitative PCRs were performed using a Real-Time Step One (Applied Biosystem, Foster City, CA, USA) by using the recommended thermal-cycling conditions.

Here, *BcSac7* (BC1G_01690, GenBank accession number XM_024693149.1) and *β-tubulin A* (*BctubA,* GenBank accession number XM_024690731.1) were selected as housekeeping genes. Although all the endogenous control genes tested exhibited stable expression among the different samples, *BctubA* was chosen to normalize gene expression data for its high transcriptional stability. The amplifications of the target genes and the endogenous controls were run using three biological replicates each with three technical replicates and were analyzed on the same plate in separate tubes. The relative abundance of transcripts was calculated by using the 2^−ΔΔ*C*^T method [53]. Relative transcripts values were calculated using control and *GFP* as reference sample. Before the quantification, a validation experiment was performed to ensure that the amplification efficiency of the target and reference genes were closely the same.

### 4.8. Off-Target Prediction

The precursor sequence used for the *BcBmp3*-dsRNA construct was targeted against the complementary DNA (cDNA) databases of some phytopathogenic and beneficial fungi (*Sclerotinia sclerotiorum*, *Alternaria alternata*, *Fusarium oxysporum*, *Rhizoctonia solani*, *Pythium ultimum*, *Trichoderma asperellum*, *T. harzianum*, *Rhizophagous irregularis*), human and lettuce using the si-Fi v21 software (default parameters) for the off-target prediction. The databases of three isolates of *B. cinerea*, including B05.10, were used as controls (Table S1).

### 4.9. In Vitro Effects of BcBmp3-Derived dsRNA on Growth of the Off-Target Fungus Trichoderma harzianum T6776

The effects of *Bmp3*-derived dsRNA (*BcBmp3*-dsRNA) on fungal growth was studied in 96-well polystyrene microtiter plates as described above for *B. cinerea* (Section 4.2). The optical density (OD) at 595 nm of fungal mycelium at different times (24, 48, 72 and 96 h) was measured.

### 4.10. Statistical Analysis

Data obtained from the in vitro assay in the 96-microtiter plates were converted as growth percentage of untreated control.

All data were subjected to analysis of variance (ANOVA) (Tukey’s HSD test for *p* ≤ 0.05) using the statistical program CoStat 6.4 (Cohort Software, Monterey, CA, USA). The percentage data were transformed into arcsine √% before ANOVA. In the expression analysis, the values are means (±SE) from three different RNA replicates for each treatment.

## Data Availability

Data are contained within the article or in supplementary materials.

## Supplementary Materials

The following are available online at https://www.mdpi.com/article/10.3390/ijms22105362/s1. Figure S1. Nucleotide sequence of the *Bmp3* gene of *Botrytis cinerea* B05.10 [1]. Figure S2. Graphical output of the predicted total and efficient siRNA hits in the *Botrytis cinerea* B05.10 *Bmp3* mRNA calculated by the si-Fi v21 software. Figure S3. *In vitro* effects of *BcBmp3*-dsRNA on *B. cinerea* B05.10 growth. Table S1. Prediction of *BcBmp3* off-target transcripts using the si-Fi v21 software. Figure S4. Graphical output of the predicted total and efficient siRNA hits in the BcBmp3 fragment used for the synthesis of dsRNA calculated by the si-Fi v21 software. Figure S5. *In vitro* effects of *BcBmp3*-derived dsRNA on *Trichoderma harzianum* T6776 growth. Table S2. Gene-specific primers used in this study. In square brackets is indicate the chromosome number of *Botrytis cinerea* B05.10 [1,2].

## Abbreviations

BcBmp3	Botrytis cinerea Slt2-type mitogen-activated protein kinase gene
BcDCL1	Botrytis cinerea Dicer-Like protein 1 (Dicer-Like gene 1)
BcDCL2	Botrytis cinerea Dicer-Like protein 2 (Dicer-Like gene 2)
CDD	Conserved Domain Database
CTRL	Control
CWI	cell wall integrity
dpi	days post inoculation
dsRNA	double-stranded RNA
GFP	Green Fluorescent Protein gene
hpRNA	hairpin RNA
MAPKs	Mitogen-activated protein kinases
non-GMO	non-Genetically Modified Organism
PTGS	Post-Transcriptional Gene Silencing
qRT-PCR	quantitative Real-Time PCR
RNAi	RNA interference
SIGS	Spray-Induced Gene Silencing
siRNA	small interfering RNA
