# Supplementary material for: Silencing of the Slt2-Type MAP Kinase Bmp3 in Botrytis cinerea by Application of Exogenous dsRNA Affects Fungal Growth and Virulence on Lactuca sativa
Source: Int J Mol Sci. 2021 May 19;22(10):5362. doi: 10.3390/ijms22105362 (PMC8161090; doi:10.3390/ijms22105362)
Supplement: Supplementary file 1 [file ijms-22-05362-s001.zip › ijms-1194989-supplementary (1).pdf]

International Journal of *Molecular Sciences*

MDPI

Supplementary Materials

Silencing of the Slt2-type MAP kinase *Bmp3* in *Botrytis cinerea* by application of exogenous dsRNA affects fungal growth and virulence on *Lactuca sativa*

Maria Spada *[1], Claudio Pugliesi [1], Marco Fambrini [1] and Susanna Pecchia *[1,2]

[1] Department of Agriculture Food and Environment, University of Pisa, Via del Borghetto 80, 56124 Pisa, Italy; claudio.pugliesi@unipi.it (C.P.); marco.fambrini@unipi.it (M.F.)

[2] Interdepartmental Research Center Nutrafood "Nutraceuticals and Food for Health", University of Pisa, Via del Borghetto 80, 56124, Pisa, Italy

* Correspondence: maria.spada@phd.unipi.it (M.S.); susanna.pecchia@unipi.it (S.P.)

ATGGCAGACCTGCAAGGAAGAAAGGTCTTCAAGGTCTTCAACCAAGACTTTATCGTCGACGAGAGATACACAGTCACCAAG
GAGCTTGGTCAAGGAGCTTACGGTATTGTTTGGTACGATGTTACTTTCCTTCCGACCCTACCTGCATATGCGCATGTCCGT
ATCATGAACCTTCAACTAACATTGTTCTCCATGGCAGTGCTGCTACGAATTCTCAAACACAGGAAGGTGTCGCAATTAAGA
AAGTTACAAATGTCTTCAGCAAGAAGATTTTGGCAAAGCGTGCGCTGCGTGAGATAAAGTTGCTACAACATTTCAGAGGTC
ATCGTAACGTATGCGACTCCTATTACACTCACGATAATGGTGGTGGGGTAGTTCTAACATGGGATAGATTACCTGCCTTTA
TGATATGGATATTCCTCGACCAGATAACTTTAATGAAACATATCTCTATGAAGGTAAGATGGGGGATTGTTTCCTGTAACT
CGTTGTTCCATGCTAATAATCTTTTCGCAGAGCTCATGGAGTGTGATCTCGCTGCTATCGTACGATCCGGTCAACCCCTCA
CTGATGCACATTTCCAATCTTTCATTTACCAAATCCTTTGCGGTCTAAAATATATTCATTCCGCGAATGTTCTCCATCGTG
ATTTGAAGCCCGGTAATTTGCTGGTCAATGCCGACTGCGAGTTGAAGATCTGTGATTTCGGTCTTGCTAGAGGTTTCTCTG
TCGACCCCGAAGAGAATGCAGGATACATGACCGAATACGTTGCTACAAGATGGTATCGTGCCCCTGAAATTATGTTGAGCT
TCCAGAGCTATACAAAAGCTAGTAAGTTATGCAACCATTGGTTGGAAAAGAAGTTATAAGCGCTGACATAAATTAGTCGAC
GTATGGTCAGTAGGATGTATTCTCGCAGAATTGCTAGGCGGTCGTCCTTTCTTCAAAGGTAGAGATTATGTTGATCAACTC
AACCAAATTTTACATATCCTTGGTACACCAAATGAAGAAACCCTTTCAAGAATTGGATCGCCACGAGCACAGGAATATGTT
CGAAACCTCCCATATATGGCGAAGCGACCTTTCCCAACTTTATTCCCCAACGCCAACCCCGACGCCCTTGACCTTCTTGAT
CATATGTTAGCCTTTGATCCATCTTCCCGTATCGATGTCGAAACTGCCCTCGAACACCCATACCTTCACATTTGGCACGAC
GCCTCTGATGAGCCTGGATGCCCAACAACATTTAACTTCGATTTTGAAGTTGTGGAAGATGTAGGCGAGATCAGAAAGTTG
ATTTTGGAAGAGGTTCACAGATTCAGACAACATGTCCGTGTTCAACCTGGTCAACAAGGCCAGAGCAACGGCCCTCAAGTA
CCTATACCACAAGAACAAGCAAGCTGGCGTGCCGAGGATCCAAGGCCACAAGAAGCTTATGGACAAGGGCCAAATGATCTT
GAACAAGATTTACAAGGTGGTTTGGATGCTATGCGATCG**TAG**

Figure S1. Nucleotide sequence of the *Bmp3* gene of *Botrytis cinerea* B05.10 [1]. The start and stop codons are in bold characters and highlighted in green and magenta, respectively. The non-coding intronic regions are highlighted in yellow. The annealing part of the primers with T7 promoter are highlighted in light blue and gray (see also Table S2).

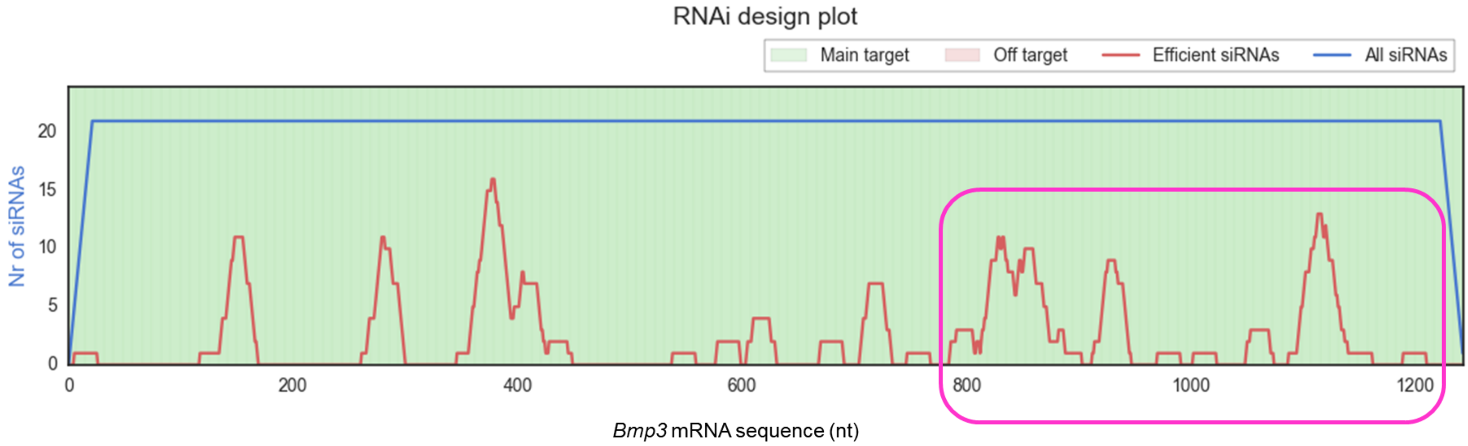

Figure S2. Graphical output of the predicted total and efficient siRNA hits in the *Botrytis cinerea* B05.10 *Bmp3* mRNA calculated by the si-Fi v21 software. The pink box includes the sequence used to generate the fragment of 427 bp for the synthesis of dsRNA (fifth exon of the gene, see Figure S1).

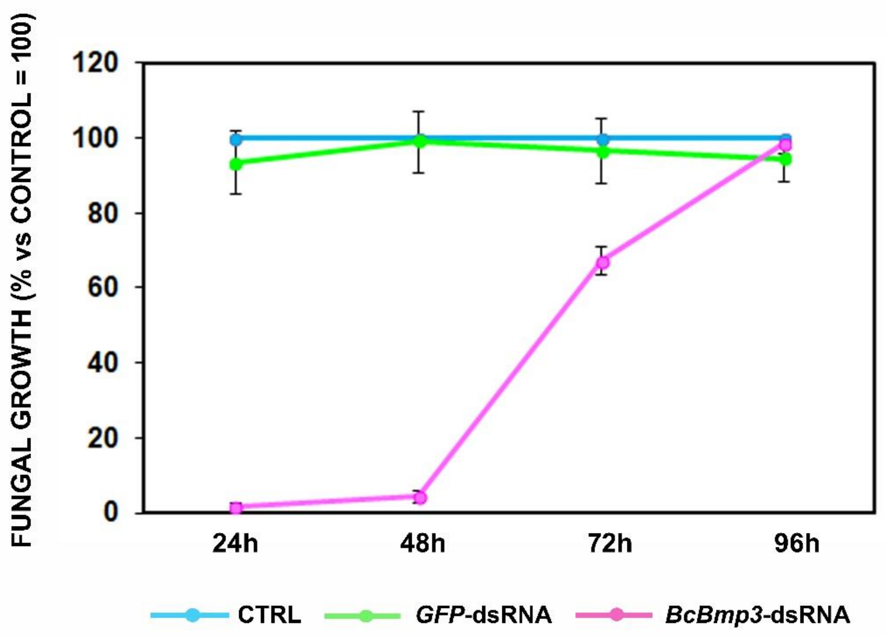

Figure S3. *In vitro* effects of *BcBmp3*-dsRNA on *B. cinerea* B05.10 growth. Fungal growth was assessed measuring the optical density (OD) at 595 nm at 24, 48, 72 and 96 hours (24h, 48h, 72h and 96h) in 96-well microtiter plates. Each well contained 500 conidia in SMB medium and 2 µg of *GFP*-dsRNA or *BcBmp3*-dsRNA. SMB + TE buffer was used as control (CTRL). The graph shows the mean (± SE) of two independent experiments with eight biological replicates (n = 8). Data obtained from the *in vitro* assay were converted as growth percentage of untreated control.

Table S1. Prediction of *BcBmp3* off-target transcripts using the si-Fi v21 software.

	All siRNA*	Efficient siRNA**
Botrytis cinerea B05.10[a]	407	210
Botrytis cinerea T4[b]	407	210
Botrytis cinerea DW1[c]	386	202
Sclerotinia sclerotiorum[d]	18	9
Sclerotinia sclerotiorum 1980 UF-70[e]	18	9
Alternaria alternata SRC1lrK2f[f]	0	0
Fusarium oxysporum[g]	0	0
Rhizoctonia solani AG-1 IA[h]	0	0
Pythium ultimum ASM14694[i]	0	0
Trichoderma asperellum CBS 433.97[l]	0	0
Trichoderma harzianum T6776[m]	0	0
Rhizophagous irregularis DAOM 197198[n]	0	0
Lactuca sativa cv. Salinas[o]	0	0
Homo sapiens (GRCh38.p13)[p]	0	0

Below the links of the cDNA gene sequence files are reported:

[a] ftp://ftp.ensemblgenomes.org/pub/fungi/release-50/fasta/botrytis_cinerea/cdna/

[b] ftp://ftp.ensemblgenomes.org/pub/fungi/release-50/fasta/fungi_ascomycota1_collection/botrytis_cinerea_t4_gca_000227075/cdna/

[c] ftp://ftp.ensemblgenomes.org/pub/fungi/release-50/fasta/fungi_ascomycota1_collection/botrytis_cinerea_bcdw1_gca_000349525/cdna/

[d] ftp://ftp.ensemblgenomes.org/pub/fungi/release-50/fasta/sclerotinia_sclerotiorum/cdna/

[e] ftp://ftp.ensemblgenomes.org/pub/fungi/release-50/fasta/fungi_ascomycota3_collection/sclerotinia_sclerotiorum_1980_uf_70_gca_001857865/cdna/

[f] ftp://ftp.ensemblgenomes.org/pub/fungi/release-50/fasta/fungi_ascomycota3_collection/alternaria_alternata_gca_001642055/cdna/

[g] ftp://ftp.ensemblgenomes.org/pub/fungi/release-50/fasta/fusarium_oxysporum/cdna/

[h] ftp://ftp.ensemblgenomes.org/pub/fungi/release-50/fasta/fungi_basidiomycota1_collection/rhizoctonia_solani_ag_1_ia_gca_000334115/cdna/

[i] ftp://ftp.ensemblgenomes.org/pub/protists/release-50/fasta/pythium_ultimum/cdna/

[l] ftp://ftp.ensemblgenomes.org/pub/fungi/release-50/fasta/fungi_ascomycota4_collection/trichoderma_asperellum_cbs_433_97_gca_003025105/cdna/

[m] ftp://ftp.ensemblgenomes.org/pub/fungi/release-50/fasta/fungi_ascomycota2_collection/trichoderma_harzianum_gca_000988865/cdna/

[n] ftp://ftp.ensemblgenomes.org/pub/fungi/release-50/fasta/fungi_mucoromycota1_collection/rhizophagus_irregularis_daom_197198w_gca_000597685/cdna/

[o] https://www.ncbi.nlm.nih.gov/assembly/GCA_002870075.2

[p] http://ftp.ensembl.org/pub/release-103/fasta/homo_sapiens/cdna/

* Number of siRNA sequences (21-mer) that perfectly match the query sequence.

** Number of siRNA sequences (21-mer) with perfect match to the query sequence that meet additional criteria for efficient RNAi.

Target	Total siRNA hits	Efficient siRNA hits
BcBmp3	407	210

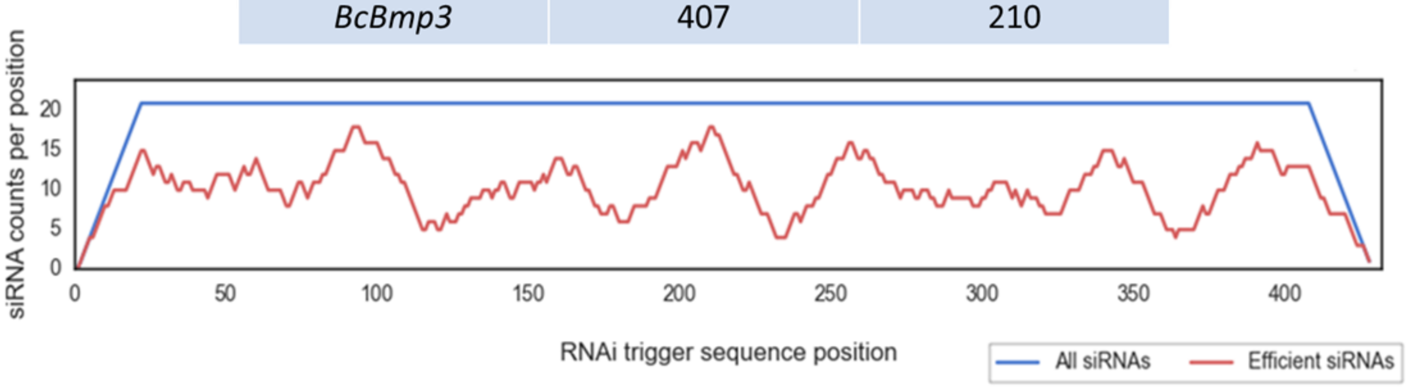

Figure S4. Graphical output of the predicted total and efficient siRNA hits in the *BcBmp3* fragment used for the synthesis of dsRNA calculated by the si-Fi v21 software.

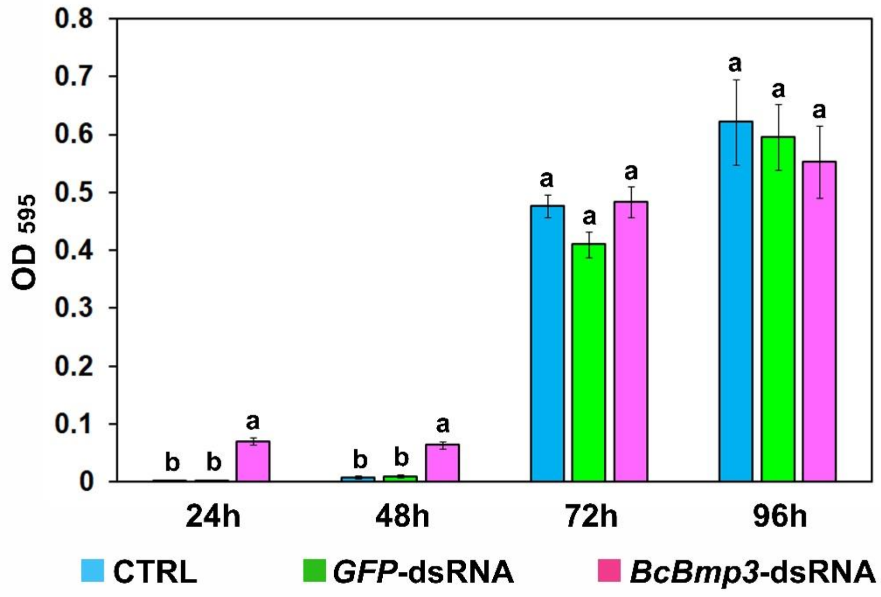

Figure S5. *In vitro* effects of *BcBmp3*-derived dsRNA on *Trichoderma harzianum* T6776 growth. Fungal growth was assessed measuring the optical density (OD) at 595 nm at 24, 48, 72 and 96 hours (24h, 48h, 72h and 96h) in 96-well microtiter plates. Each well contained 500 conidia in SMB medium and 2 µg of *GFP*-dsRNA or *BcBmp3*-dsRNA. SMB + TE buffer was used as control (CTRL). The graph shows the mean (± SE) of two independent experiments with eight biological replicates ($n = 8$). Same letters above the bars indicate no significant differences from each other (ANOVA) according to Tukey's test ($p \leq 0.05$). The statistical analysis was conducted separately for 24, 48, 72 and 96 hours.

Table S2. Gene-specific primers used in this study. In square brackets is indicate the chromosome number of *Botrytis cinerea* B05.10 [1,2].

**Use**	**GenBank accession number**	**Primer**	**Primer sequence 5′-3′ (F: Forward; R: Reverse)**	**Amplicon size (bp)**
Amplification of the *Bmp3* gene fragment from *Botrytis cinerea* (*BcBmP3*) [BCIN09] for *in vitro* transcription	CP009813.1	Bmp3T7F<br>Bmp3T7R	F:TAATACGACTCACTATAGGGAGACCACGAGCACAGGAATATG<br>R:TAATACGACTCACTATAGGGAGACAAGATCATTTGGCCCTTGT	427
Amplification of the *GFP* gene fragment from plasmid pCT74-sGFP for *in vitro* transcription		GFP1T7F<br>GFP1T7R	F:TAATACGACTCACTATAGGGAGAGTGAGCAAGGGCGAG<br>R:TAATACGACTCACTATAGGGAGATTGTACAGCTCGTCCAT	712
Amplification by qRT-PCR of the *Bmp3* gene of *Botrytis cinerea* (*BcBmp3*) [BCIN09]	CP009813.1	9F<br>8R	F:AGAATTGCTAGGCGGTCGTC<br>R:AACATATTCCTGTGCTCGTGGC	136
Amplification by qRT-PCR of the housekeeping gene *beta tubulin A* of *Botrytis cinerea* (*BctubA*) [BCIN01]	XM_024690731.1	18F<br>19R	F:GTCTCAAGATGTCCTCCACC<br>R:ACTCCATCTCGTCCATACCT	143
Amplification by qRT-PCR of the housekeeping gene *Sac7* of *Botrytis cinerea* (*BcSac7*) [BCIN05]	XM_024693149.1	50F<br>51R	F:CTCAGTGGCTCGGAAAAGC<br>R:CGTTTGCCGCATCATGAACTG	112

References

1. Amselem, J.; Cuomo, C.A.; van Kan, J.A.; Viaud, M.; Benito, E.P.; Couloux, A.; Coutinho, P.M.; de Vries, R.P.; Dyer, P.S.; Fillinger, S.; et al. Genomic analysis of the necrotrophic fungal pathogens *Sclerotinia sclerotiorum* and *Botrytis cinerea*. *PLoS Genet.* **2011**, *7*, e1002230, doi:10.1371/journal.pgen.1002230.
2. Van Kan, J.A.; Stassen, J.H.; Mosbach, A.; Van Der Lee, T.A.; Faino, L.; Farmer, A.D.; Papasotiriou, D.G.; Zhou, S.; Seidl, M.F.; Cottam, E.; et al. A gapless genome sequence of the fungus *Botrytis cinerea. Mol. Plant Pathol.* **2017**, *18*, 75-89, doi:10.1111/mpp.12384.
